# Spinopelvic alignment predicts disc calcification, displacement, and Modic changes: Evidence of an evolutionary etiology for clinically‐relevant spinal phenotypes

**DOI:** 10.1002/jsp2.1083

**Published:** 2020-02-19

**Authors:** Uruj Zehra, Jason P. Y. Cheung, Cora Bow, Rebecca J. Crawford, Keith D. K. Luk, William Lu, Dino Samartzis

**Affiliations:** ^1^ Department of Anatomy University of Health Sciences Lahore Pakistan; ^2^ Department of Orthopaedics and Traumatology The University of Hong Kong Pokfulam Hong Kong; ^3^ Faculty of Health Sciences Curtin University Perth Australia; ^4^ Department of Orthopaedic Surgery RUSH University Medical Center Chicago Illinois USA; ^5^ International Spine Research and Innovation Initiative RUSH University Medical Center Chicago Illinois USA

**Keywords:** incidence, lordosis, lumbar, Modic, MRI, pelvic, sacral slope, spinopelvic alignment, tilt, ultra‐short, UTE

## Abstract

Lumbar disc‐displacement, Modic changes (MCs), and UTE Disc Sign (UDS) on MRI are clinically relevant spinal phenotypes that can lead to sciatica/LBP. Not all degenerated discs result in disc‐displacement, MCs and UDS, suggesting varied etiologies. Spinopelvic parameters have been implicated in various spinal disorders. Pelvic incidence (PI) is “fixed parameter” since skeletal maturity. No study has addressed disc‐displacement, MCs and UDS in context of spinopelvic parameters. Therefore, the aim of study was to determine if spinopelvic parameters are associated and predict clinically‐relevant MRI‐phenotypes. One hundred and eight population‐based subjects (mean age: 52.3 years) were recruited. Spondylolisthesis and scoliosis individuals were excluded. Lumbar lordosis (LL), PI, sacral slope (SS), and pelvic tilt (PT) were assessed on lateral plain radiographs. Disc degeneration was assessed and summated, and presence or not of disc‐displacement and MCs were noted on T2W MRI. UDS was detected on UTE. Following exclusion criteria, 95 subjects were assessed. Disc‐displacement (82.1%), MCs (52.6%), and UDS (37.9%) were associated with lower PI, SS, LL, and LL/PI index. On multivariate analyses, lower PI was significantly related to development of these MRI phenotypes (adjusted OR range:0.95‐0.92; *P* < .05), with critical PI value of 42° or lower exhibiting fourfold increase risk of combined phenotypes (*P* = .020). Of UDS discs, 39.3% had adjacent MCs and 83.6% had disc‐displacement. 87.5% of MC had directly adjacent UDS. The first study to note that PI may “predict” the development of disc‐displacement, MCs and UDS, suggesting potential sub‐variants and mechanistic susceptibility that may be grounded in spinopelvic evolution. An “evolutionary etiological pathway” of spinal phenotype development is proposed.

## Graphical abstract

Our findings suggest a strong biomechanical influence of spinopelvic morphometry on clinically meaningful anterior column spinal phenotypes, which may have an etiological foundation in spinal evolution secondary to permanent bipedalism. Our results improve the understanding of spinal phenotypes, contributing additional steps toward personalized spine care.

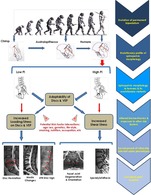

## 1 INTRODUCTION

Low back pain (LBP) is the leading disability worldwide and a tremendous socioeconomic burden.^1^, ^2^ Mismatches between conventional T2‐weighted (T2W) MRI‐defined disc degeneration and LBP exist.^3^, ^4^ Spinal phenotypes consistently associated with, and predictive for, LBP and/or sciatica, are lumbar disc displacement and Modic changes (MCs; ie, subchondral vertebral bone marrow lesions).^4^, ^5^ Vertebral endplate structural defects, such as Schmorl's nodes, may often be benign.^6^ Lifestyle factors, excessive loading, and genetics among others may contribute to the development of such spinal phenotypes.^5^, ^7^, ^8^ However, such risk factors are often not consistently replicated, suggesting that other factors can be more influential and/or part of the spectrum.^9^, ^10^

The “UTE Disc Sign” (UDS) on ultra‐short time‐to‐echo (UTE) MRI by Pang et al^11^ was recently reported, and found to be strongly associated with disc degeneration severity, lumbar disc displacement, MCs, LBP, and disability. The UDS was noted to may represent disc calcification, which usually embody active inflammation and can “stiffen” the disc material; thereby, affecting the kinematics of the disc and motion segment.^12^ Such calcification has been found in degenerative and scoliotic discs, suggesting that abnormal mechanical loading may be a likely mechanism.^13^ Therefore, the UDS may potentially have a role in the initiation and propagation of lumbar disc displacement and/or instability, causing disc and endplate disruptions/damage that can potentially lead to MCs; thereby, increasing the risk for LBP.

**Figure 1:**
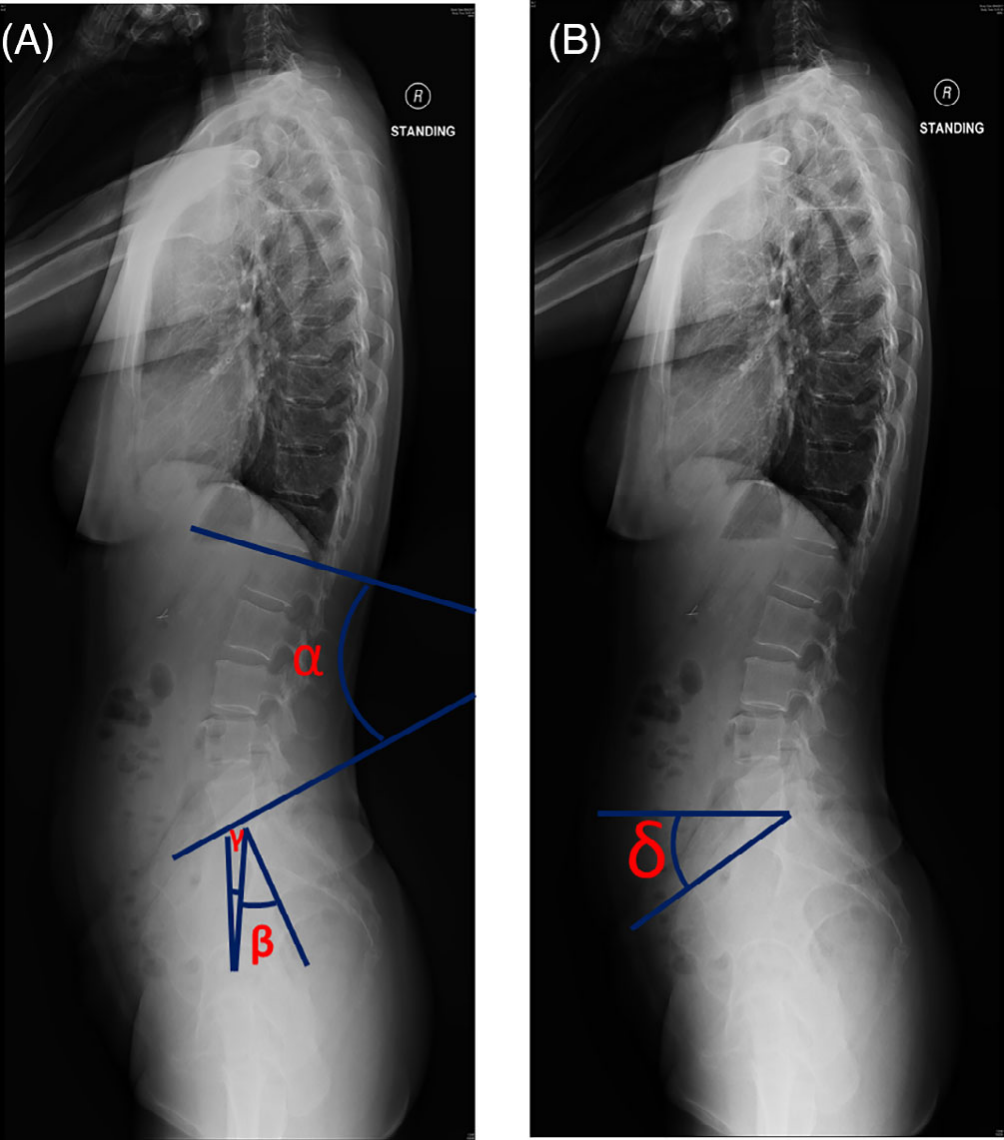
Representative plain radiographic lateral images of the full spine illustrating measurement of (A) lumbar lordosis (*α*): the resultant angle of the line intersecting the superior endplate of L1 and the inferior endplate of L5, pelvic incidence (*β*): the resultant angle of the line perpendicular to the superior sacral endplate and the line connecting the midpoint of the superior sacral endplate to the mid‐point of femoral head axis, pelvic tilt (*γ*): the resultant angle of the line connecting the midpoint of the sacral plate to the mid‐point of the femoral head axis and the vertical plane. (B) Sacral slope (*δ*): the resultant angle between the superior plate of S1 and the horizontal line

Balanced sagittal alignment of the spine is vital for spinal function and is crucial to maintain upright postures.^14^, ^15^ The neutral upright sagittal alignment is achieved only when the spine and pelvis are in sync.^16^, ^17^ The harmonious connection of the pelvis with the spine, also known as “spinopelvic balance,” contributes considerably to overall sagittal balance. The three main defining spinopelvic parameters are *pelvic incidence (PI)*, *pelvic tilt (PT)*, *and sacral slope (SS)* (Figure 1).^18^

PI is a “fixed” parameter, akin to one's genetic make‐up, at skeletal maturity^19^ and may vary between individuals.^14^ PI is essentially an individual's genetic blue‐print. This is based on concurrent spinopelvic adaptations as a consequence of human evolutionary requirements for a well‐balanced upright spine in the progression toward permanent bipedalism.^20^ Increased PI is an adaptation for naturally selecting a nearly perfect pelvis that can support the upright trunk and at the same time provide energy‐efficient bipedalism.^21^ PT and SS are usually determined by pelvic orientation and can vary.^17^

PI, a summation of the PT and SS, is arguably the most studied spinopelvic parameter^22^ and may highly be associated with pain and disability.^15^, ^17^, ^23^ PI directly correlates with lumbar lordosis (LL),^24^ which may be affected by degenerative changes.^17^ PI is generally thought to be unaffected by lumbar degenerative changes.^17^ Association of increased PI with spondylolisthesis^25^ and facet joint degeneration^14^, ^16^ has been reported by several studies. Previously, a decreased PI was noted with degenerative disc disease, lumbar disc displacement, and chronic LBP.^17^, ^26^, ^27^ Therefore, spinopelvic balance disruption can alter biomechanical stresses at the lumbo‐sacral junction and also in the compensation mechanisms used to maintain an adequate posture,^28^ leading to aforementioned spinal and disc changes as well as LBP. However, not all disc degeneration on MRI may lead to lumbar disc displacement and/or MCs, and that potential sub‐variants in the etiologies of these phenotypes may exist.^5^

Since the advent of MRI, a discrepancy still remains between various spinal phenotypes and their potential etiological factors.^29^, ^30^, ^31^ Therefore, it is very plausible that spinopelvic parameters may be the “missing link” between clinically‐relevant phenotypes, such as lumbar disc displacement, MCs, and UDS, and their development. Therefore, such spinopelvic parameters warrant further investigation to address this constellation of MRI findings. As such, this study aimed to determine if spinopelvic parameters, in particular PI (fixed entity), are associated and can “predict” the development of clinically‐relevant MRI phenotypes of lumbar disc displacement, MCs, and UDS.

## 2 METHODS

Study design: Cross sectional.

Level of evidence: II.

### 2.1 Study population

One hundred and eight Southern Chinese volunteers with age range of 22 to 67 years (mean age: 52 years; 50% males) were recruited from a population study to be part of a new cohort addressing novel imaging. The recruitment parameters have been reported elsewhere.^7^, ^8^, ^32^ In short and following institutional review board approval, 108 subjects were randomly recruited, irrespective of pain profile. No subjects underwent previous spine surgery. The sample size was determined based on funding constraints; however, currently represents the first study to date that have undergone T2‐ and T1‐weighted as well as UTE MRI along with standing lateral plain radiographs. All subjects were enrolled consecutively and informed consent was obtained. Of these 108 subjects, 11 subjects were excluded for the following study because they exhibited spondylolisthesis, scoliosis, trauma, or active infections on MRI that were also confirmed on plain radiographs, and/or the femoral head was not visible to facilitate PI assessment. Two subjects were also excluded because, due to scheduling conflicts, did not undergo plain radiograph assessment. As such, 95 subjects were included in the following cross‐sectional study.

### 2.2 Radiographic measurements

Lateral plain standing radiographs of the lumbar spine (L1‐S1), pelvis and proximal femur were assessed. The volunteers were standing erect with arms raised and slightly fisted hands resting on their clavicles. The film focus distance was 180 cm and other exposure factors were 88 kVp and 32 mAS. These radiographic acquisition parameters were kept consistent in all the individuals, irrespective of their body mass index (BMI). All radiographs were acquired digitally. LL and spinopelvic parameters (ie, PI, PT, and SS) were measured based upon the lateral radiographs, and are defined as illustrated in Figure 1.^15^, ^17^ The LL/PI index was also tabulated.^33^ A medical doctor experienced in image assessment and blinded to MRI findings assessed all plain radiographs (UZ). All radiological parameters on 25 randomly selected radiographs were reassessed after 3 weeks to obtain the intra‐observer reliability estimate. Based on Cronbach's alpha reliability assessment,^34^ the reliability was good to excellent (LL: *a* = 0.85, PI: *a* = 0.87, PT: *a* = 0.92, and SS: *a* = 0.91).

### 2.3 MRI Assessment

All subjects underwent MRI of L1‐S1 via a 3 T MRI scanner (Achieva, Philips Healthcare, Best, The Netherlands). Sagittal T2W MRI were acquired using a standard spin‐echo imaging sequence with the following parameters: FoV = 200 mm, slice thickness = 2.4 mm, acquisition matrix = 400 × 232, and TE/TR = 120 ms/2000 ms. UTE MRI was acquired by a 3D UTE shifting TE phase‐encoded stack of spirals trajectory. The UTE imaging parameters were as follows: FOV = 240 mm, slice thickness = 1.2 mm, TR = 4.8 ms, TE =0.140 ms, and acquisition voxel size =0.5 × 0.5mm^2^. The axial T1W sequences obtained for this cohort were not employed.

Based on sagittal T2W MRI, the Pfirrmann et al^35^ grading system was used to assess disc degenerative scores. Grades 4 and 5 were regarded as “black discs.” A cumulative “disc degeneration score” (potential range: 0‐25)^36^ was obtained from a summation of individual discs scored from L1 to S1 via the Pfirrmann et al^35^ method. Lumbar disc displacement (disc bulges, protrusion, and extrusion were grouped together), represented by the displacement of the annular fibers beyond the vertebral margin were assessed. MCs^10^, ^37^ were noted, regardless of the Type due to the study sample size. One may deduce that the majority of the MCs were Type II, as noted in our previous studies of this ethnic group population.^37^ In fact, we have found Modic Type I and Type II to be associated with pain/disability in past studies.^10^, ^54^ Sagittal UTE MRI was used to detect UDS (ie, hypo‐intense disc band).^11^ The location of the UDS in relation to MCs and lumbar disc displacement was also noted. If UDS and MCs overlapped or not in the same location, this observation was noted. MRI phenotypes were assessed by trained raters (U.Z., J.P.Y.C., and D.S.). All raters were kept blinded to radiographic findings. The inter‐ and intrarater reliability of T2W MRI and UTE MRI phenotypes were excellent (*k* = 0.91, *k* = 1.00, respectively) and previously reported.^11^, ^32^ Age (years), sex‐type (males vs females), body weight (kilograms), body height (meters), and BMI (kg/m^2^) were noted for every individual.

### 2.4 Statistical analyses

SPSS v24 (Chicago, Illinois) was used to perform the statistical analyses. Descriptive and frequency analyses were performed of the data set, noting percent (%), and mean ± SD (SD) values. The spinopelvic parameters were found to be parametric. Univariate analyses consisted of independent‐samples *t*‐test and chi‐square or Fisher's Exact Test where appropriate. Pearson correlation analysis (*r*) was obtained. Multivariate analyses consisted of binary logistic regression, whereby odds ratios (OR) and 95% confidence intervals (CI) were evaluated. Due to the sample size, a limit was placed as to how many co‐variates could be used in a model. For the context of this study and based on prior knowledge, age, sex‐type, and BMI were the subject characteristics noted in each model, along with the PI and the PI‐LL index since the PI parameter is the only spinopelvic measure that is “fixed” in individuals and nonmodifiable to assess “prediction.” The cumulative disc degeneration score was also used for model adjustment in an attempt to control for potential disc degeneration severity effects upon the development of lumbar disc displacement, MCs, UDS and a combination of all three. Factors related to black discs were also assessed. Statistical significance was established at *P* < .05.

## 3 RESULTS

Following exclusion of 13 subjects, 95 subjects were included in the study. There were 45 males (47.4%) and 50 (52.6%) females, with a mean age in years of 52.4 (SD:7.4) and a mean BMI in kg/m^2^ of 24.6 (SD:3.6). There were 69 (72.6%) subjects with black discs, 78 (82.1%) with lumbar disc displacement, 50 (52.6%) with MCs, 36 (37.9%) with UDS, and 23 (24.2%) with combined lumbar disc displacement /MCs/UDS. The cumulative disc degeneration score of L1‐S1 was 15.9 (SD:3.0). The number of disc levels (range from 0 to 5) with lumbar disc displacement, MCs and UDS were 2.4 (SD:0.7), 1 (SD:1.3), and 0.6 (SD:1.0), respectively. The mean values in degrees of PI, SS, PT, LL, and LL/PI index were 45.1 (SD:11.6), 28.2 (SD:7.1), 11.6 (SD:8.0), 28.6 (SD:12.1), and 0.64 (SD:0.27), respectively. Age, BMI and cumulative disc degeneration scores were not found to significantly correlate with the spinopelvic parameters (Table 1). Table 2 illustrates the univariate analyses of the spinopelvic parameters in relation to the different phenotypes.

**Table 1:** Correlation analyses between subject demographics and cumulative degenerative disc score of L1‐S1 on magnetic resonance imaging (MRI) to that of spinopelvic parameters

	Variables		
Spinopelvic parameters	Age	BMI	Cumulative Pfirrmann disc degeneration score
	(years)	(kg/m^2^)	
Pelvic incidence (degrees)	*r* = .184, *P* = .75	*r* = .014, *P* = .898	*r* = .183, *P* = .076
Sacral slope (degrees)	*r* = .161, *P* = .119	*r* = −.081, *P* = .447	*r* = .123, *P* = .234
Pelvic tilt (degrees)	*r* = .026, *P* = .806	*r* = −.060, *P* = .571	*r* = .159, *P* = .124
Lumbar lordosis (degrees)	*r* = .109, *P* = .293	*r* = .058, *P* = .587	*r* = −.076, *P* = .465
Lumbar lordosis/pelvic incidence index	*r* = .018, *P* = .865	*r* = .100, *P* = .345	*r* = −.153, *P* = .139

Abbreviations: BMI, body mass index; kg, kilograms; m, meters; UTE: ultra‐short time‐to echo.

**Table 2:** Univariate association of various lumbar spinal phenotypes on T2‐weighted magnetic resonance imaging (MRI) and ultra‐short time‐to echo (UTE) MRI to that of spinopelvic parameters

	Lumbar phenotypes on MRI									
	Disc degeneration (black disc)		Disc displacement		Modic changes		UTE disc sign^a^		Combined disc displacement, Modic changes, and UTE disc sign	
Spinopelvic parameters	No *n* = 26	Yes *n* = 69	No *n* = 17	Yes *n* = 78	No *n* = 45	Yes *n* = 50	No *n* = 59	Yes *n* = 36	No *n* = 72	Yes *n* = 23
Pelvic incidence (degrees)	43.5 ± 11.0	45.7 ± 11.8 *P =* .420	47.4 ± 12.7	44.6 ± 11.4 *P =* .366	46.8 ± 12.0	43.5 ± 11.1 *P =* .171	46.4 ± 12.5	43.2 ± 9.9 *P =* .228	46.1 ± 11.9	41.9 ± 1.1 *P =* .127
Sacral slope (degrees)	27.6 ± 6.8	28.5 ± 7.3 *P =* .566	32.2 ± 8.1	27.4 ± 6.68 *P =* .010*	3.0 ± 7.9	26.7 ± 5.99 *P =* .025*	28.2 ± 6.9	28.6 ± 7.6 *P =* .991	29.1 ± 7.5	25.4 ± 5.3 *P =* .028*
Pelvic tilt (degrees)	9.9 ± 9.4	12.2 ± 7.3 *P =* .207	1.3 ± 11.9	11.9 ± 6.9 *P =* .460	1.8 ± 8.5	12.2 ± 7.5 *P =* .410	11.7 ± 8.3	11.4 ± 7.5 *P =* .890	11.4 ± 8.2	12.0 ± 7.3 *P =* .763
Lumbar lordosis (degrees)	28.0 ± 11.2	28.8 ± 12.5 *P =* .762	35.6 ± 1.0	27.0 ± 12.1 *P =* .007*	31.5 ± 11.8	25.8 ± 11.9 *P =* .021*	3.0 ± 11.6	26.2 ± 12.8 *P =* .141	3.2 ± 11.9	23.6 ± 11.6 *P =* .023*
Lumbar Lordosis/pelvic incidence index	.64 ± .22	.65 ± .29 *P =* .940	.79 ± .27	.62 ± .26 *P =* .012*	.69 ± .26	.60 ± .28 *P =* .116	.66 ± .24	.62 ± .32 *P =* .521	.66 ± .26	.58 ± .30 *P =* .196

*Note*: Endplate abnormalities represent structural endplate changes/defects involving the bony/cartilaginous endplates. The value of “*n*” represents the sample size of subjects. Values are presented as mean and SD.

a Ultra‐short time‐to echo (UTE) MRI to that of spinopelvic parameters.

*P < .05.

**Figure 2:**
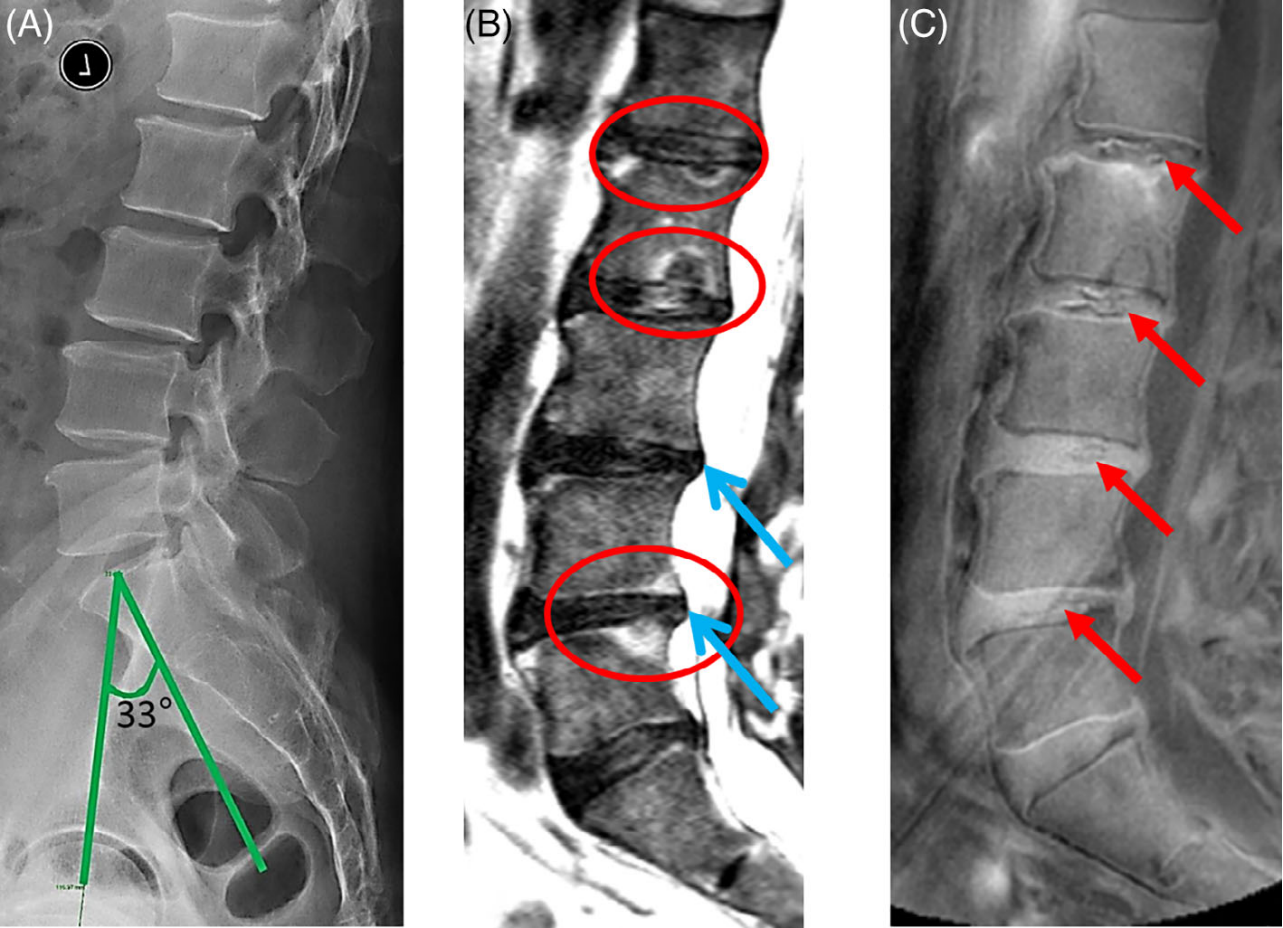
Images illustrating the lumbar spine of a 56 year‐old male by three different imaging techniques. A, Lateral plain radiograph showing low pelvic incidence of 33°. B, T2 weighted MRI scan showing Modic changes (red circles) and disc displacement (blue arrow). C, Ultra‐short time‐to‐echo (UTE) showing the UTE Disc Signs (UDS) at multiple levels (red arrows). Note the overlap of Modic changes and UDS

Of all the discs from 95 subjects, 61 discs showed UDSs. 83.6% of UDS discs were noted to have lumbar disc displacement. In 39.3% of discs, adjacent MCs were observed. MCs were mapped according to their location on the endplates (ie, anterior, middle, and posterior). Similar mapping was done for presence of the UDS in the discs. Overlapping of both phenotypes at the same site was observed in 87.5% of the discs. All discs with UDS and adjacent MCs also had lumbar disc displacement (Figure 2).

**Figure 3:**
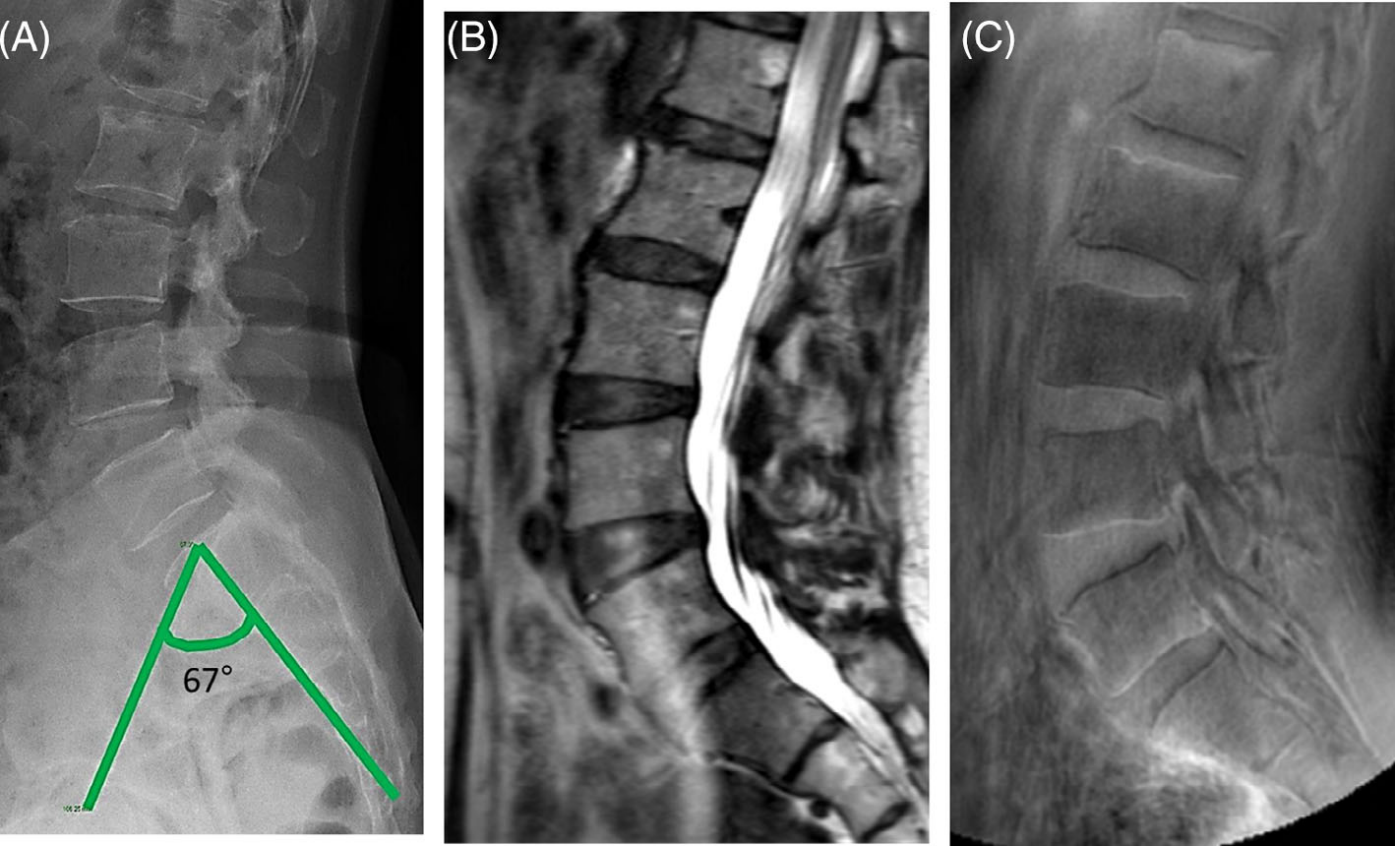
Images depicting the lumbar spine of 53 year‐old female by three different imaging techniques. A, Lateral plain radiograph showing high pelvic incidence of 67°. B, T2‐weighted MRI showing no Modic changes. C, Ultra‐short time‐to‐echo (UTE) with no UTE disc signs

Based on multivariate modeling, increasing age was the only predictive factor for the overall presence of a black disc (adjusted OR:1.17; 95% CI:1.06‐1.29; *P* = .002; Table 3). With respect to the development of lumbar disc displacement, MCs, UDS, and a combination of these three phenotypes, all models were equally adjusted for subject demographics and cumulative disc degeneration scores. For lumbar disc displacement, a lower LL/PI index (adjusted OR:0.02; 95% CI:0‐0.64; *P* = .027) and decrease PI (adjusted OR:0.93; 95% CI:0.87‐0.97; *P* = .039) were found significant. With respect to MCs (adjusted OR:0.94; 95% CI:0.90‐0.99; *P* = .025), UDS (adjusted OR:0.95; 95% CI:0.90‐0.99; *P* = .019) and the combined phenotypes (adjusted OR:0.92; 95% CI:0.87‐0.98; *P* = .009), a lower PI was significantly predictive. Adopting a conservative estimate of a PI of 42° based on the mean distribution of the combined phenotypes, a fourfold increase risk in developing these phenotypes was found in subjects who had a 42° PI or lower (adjusted OR: 4.15; 95% CI: 1.25‐13.83; *P* = .020; Figures 2 and 3).

**Table 3:** Multivariate logistic regression analysis addressing determinants of the overall presence of spinal phenotypes on T2‐weighted magnetic resonance imaging (MRI) and ultra‐short time‐to echo (UTE) MRI

	OR	95% confidence interval	*P*‐value
*Presence of disc degeneration (black disc)*			
Age (years)	1.17	1.06–1.29	.002*
Sex‐type (males)	0.52	0.16‐1.70	.277
BMI (kg/m^2^)	1.19	1.00‐1.43	.056
Lumbar lordosis/Pelvic incidence index	0.96	0.12‐7.90	.971
Pelvic Incidence (degrees)	1.02	0.96–.1.08	.554
*Presence of disc displacement*			
Age (years)	1.03	0.91‐1.17	.653
Sex‐type (males)	1.23	0.34‐4.53	.751
BMI (kg/m^2^)	0.99	0.84‐1.17	.910
Cumulative Pfirrmann Disc degeneration score	1.23	0.97‐1.56	.096
Lumbar lordosis/pelvic incidence index	0.020	0–0.64	.027*
Pelvic incidence (degrees)	0.93	0.87–0.97	.039*
*Presence of Modic changes*			
Age (years)	0.86	0.78‐0.96	.007*
Sex‐type (males)	2.97	0.95‐9.32	.063
BMI (kg/m^2^)	0.89	0.75‐1.05	.172
Cumulative Pfirrmann disc degeneration score	1.68	1.30‐2.18	<.001*
Lumbar Lordosis/pelvic incidence index	0.66	0.10‐4.35	.665
Pelvic incidence (degrees)	0.94	0.90–0.99	.025*
*Presence of UTE disc sign*			
Age (years)	1.03	0.94‐1.14	.504
Sex‐Type (Males)	1.23	0.43‐3.65	.704
BMI (kg/m^2^)	0.93	0.86‐1.16	.934
Cumulative Pfirrmann disc degeneration score	1.52	1.18‐1.96	.001*
Lumbar lordosis/pelvic incidence index	0.67	0.11‐4.24	.667
Pelvic incidence (degrees)	0.95	0.90–.991	.019*
Presence of combined disc displacement, modic changes, and UTE disc sign			
Age (years)	0.94	0.83‐1.06	.300
Sex‐type (males)	1.43	0.42‐4.84	.572
BMI (kg/m^2^)	0.98	0.82‐1.18	.865
Cumulative Pfirrmann disc degeneration score	1.84	1.29‐2.63	.001*
Lumbar lordosis/Pelvic incidence index	0.38	0.05‐2.66	.326
Pelvic incidence (degrees)	0.92	0.87‐0.98	.009*

*Note*: Cumulative Pfirrmann disc degeneration score consists of the combined individual disc scores from L1 to S1. This covariate was note used in the model addressing “Presence of Disc Degeneration (Black Disc)” since disc degeneration was a dependent variable.

Abbreviations: BMI, body mass index; kg, kilograms; m, meters.

*P < .05.

## 4 DISCUSSION

This is the first study that has assessed the predictive role of PI in lumbar disc displacement, MCs, and UDS. Our findings indicate that low PI “predicts” these clinically relevant spinal phenotypes, increasing the risk of development up to 8% for “each” decreased degree of angulation. This finding is irrespective of age, sex‐type, BMI, and disc degeneration severity. In fact, in the context of these subject demographics, PI was a greater and more significant predictive factor. A critical PI value was noted; whereby, a PI of 42° or lower exhibited a fourfold increased risk in the development of these phenotypes, this 42° was determined from ROC analyses. Several studies have investigated spinopelvic alignment in patients having certain signs of disc degeneration,^38^ lumbar disc displacement,^39^ and spondylolisthesis.^17^ However, and in particular, excluding cases of spondylolisthesis and having a more robust definition of disc degeneration in our study, we noted the novel finding that PI was significantly related to MCs, UDS as well as lumbar disc displacement and the combination of the three phenotypes. Furthermore, age and BMI were not associated with any of the pelvic parameters, which were in agreement with many previous reports.^40^, ^41^, ^42^

**Figure 4:**
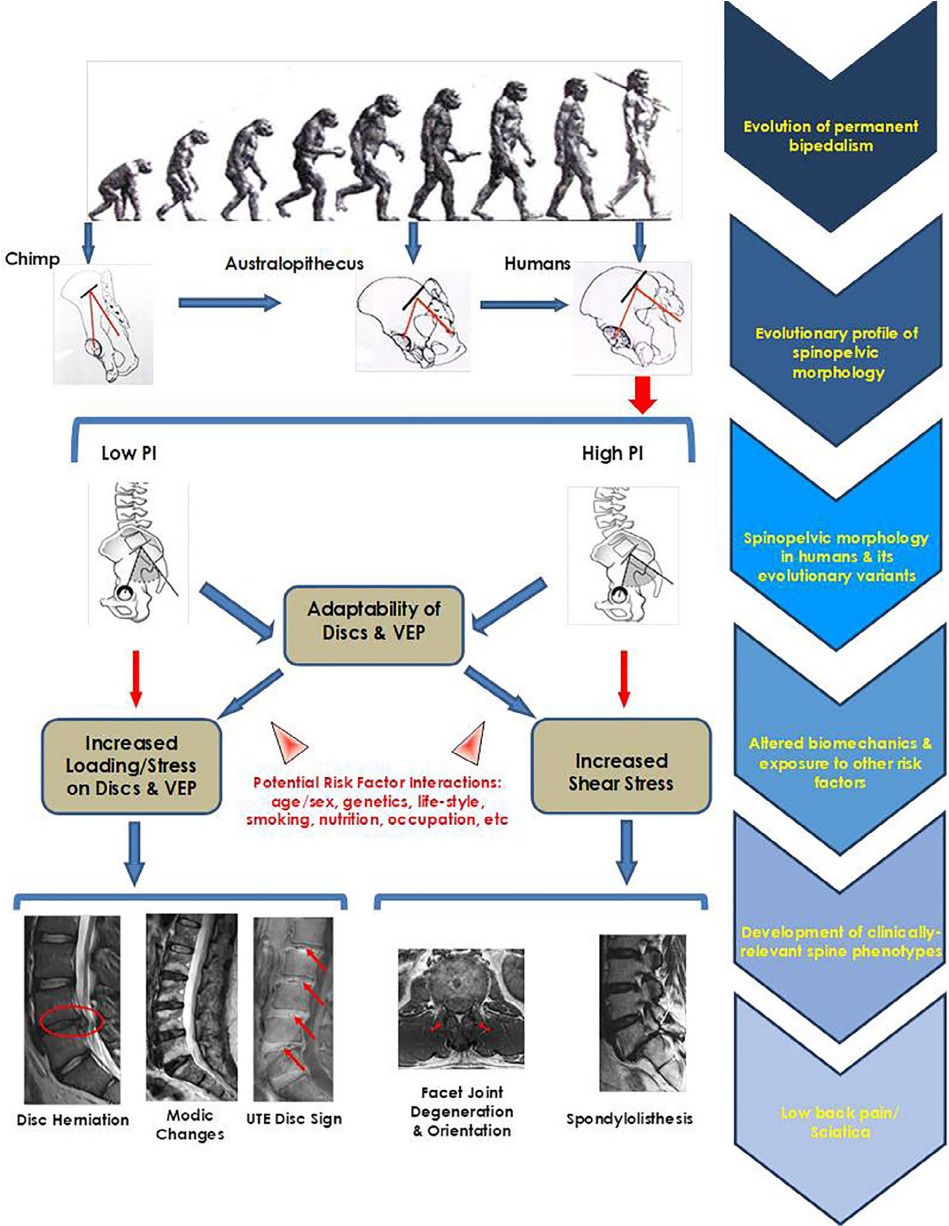
The Evolutionary Etiology pathway of the development of various spinal phenotypes

Lumbar disc displacement subjects tended to have lower PI, SS, and LL, irrespective of demographics and degree of disc degeneration. These findings are in accordance with some previous reports.^17^, ^39^ A lower PI with smaller SS may represent a more “vertical sacrum.” Moreover, a straighter spine with a less pronounced LL may exhibit greater compressive forces on the discs that may fail to adapt, which may accelerate the degeneration of the disc; thereby, producing structural failure that can lead to lumbar disc displacement and other phenotypes (Figure 4).^39^

In our study, the overlap of the UDS with MCs is a substantial step forward in understanding the significance of these disc signs.^11^, ^12^ This pattern of association indicated that altered disc integrity brought upon by disc calcification can act as a focal stressor and result in a reactive response toward the adjacent endplate that may, in some individuals, lead to MCs. Interestingly, the most convincing association was seen in individuals having UDS, lumbar disc displacement and MCs with low PI. A lower PI would alter the transmission of mechanical loading causing much of the axial compressive stresses on the discs and their adjacent vertebral endplates,^43^ supporting the development of UDS, lumbar disc displacement and associated MCs. Altered mechanical loading may increase levels of Collagen type X,^44^ which implies a positive role in cartilage calcification.^45^ The calcification process causes the gradual loss of cartilaginous endplate and reduction of disc nutritional pathways, further initiating degeneration.^46^ The degenerated discs are closely related to reduced mechanical pressure and are characterized by increased porosity and thinning of the endplates, making them vulnerable to damage.^47^ The damaged endplate removes the barrier between the discs and subchondral bone marrow, encouraging “cross‐talk” and the cascade leading to MCs.^48^ In our study, the association of lumbar disc displacement, MCs, and UDS with low PI, independent of other factors, indicates that sagittal spinopelvic alignment may substantially impact upon the development of spinal phenotypes critical to discogenic and vertebrogenic related LBP. Also, via the spinopelvic pathway, there can be variants or sub‐types within each spinal phenotype that are more attributed to such developmental/evolutionary influences and others to extraneous risk factors. Additional studies are needed to further determine the derivation of specific phenotypic patterns and their proclivity to being more symptomatic than others.

### 4.1 Evolutionary etiology pathway of spinal phenotypes

Individuals with less structural adaptation to bipedalism, such as a lower PI and vertical pelvis, are prone to sub‐optimal biomechanics and altered disc‐endplate stressors (Figure 4).^13^, ^44^, ^46^, ^47^, ^48^ Individuals with a low PI exhibit a more vertical pelvis closer to the morphology of big primates that possess a very short (anterior–posterior diameter) pelvic ring.^23^ In our “earliest upright ancestors,” bipedal walking was facilitated by some fundamental pelvic alterations.^49^ The straight alignment with small spinal curves and small PI has been identified in Neanderthal lineage hominins^20^, ^50^; interestingly, a similar morphology was seen in almost 7.8% of healthy adult modern humans.^50^ Despite evolution, variations in spinopelvic parameters in contemporary hominids may exist, contributing to their “personalized” evolutionary PI profile.

The role of genetics to the development of spinal degenerative phenotypes has been widely studied; however, heritability estimates vary from 26% to 77%.^9^, ^51^, ^52^ Considering the potential possibility of genetic predisposition to PI, we can assume that genetics may play its part in the development of disc degenerative features by determining the PI and shape of the pelvis; thereby, degenerative changes of the disc and endplate region are induced rather than sole contribution from the disc degenerative process. As such, we propose an “Evolutionary Etiology” pathway with respect to clinically‐relevant anterior column phenotypes **(**Figure 4 **)**.

### 4.2 Strengths and limitations

Like any clinical study, ours has limitations. The sample size of our study consisted of 95 individuals and parameters of back pain/sciatica could not be included in data analysis. However, to our knowledge this study represents the only study to date addressing various spinal phenotypes on conventional MRI, UTE MRI and plain radiographic parameters of spinopelvic alignment. Multivariate modeling was used to control for any potential confounders. LL can be a by‐product of spine changes; therefore, LL‐PI index, which is believed to be a statistically validated parameter, was taken into account in this model. The inclusion of stable parameter that is PI also covered PT & SS as they directly correlated with the PI angle. We further excluded cases of spondylolisthesis and scoliosis that may further confound our findings. The use of UTE MRI to assess additional spinal phenotypes that may have been hidden on traditional T2‐weighted MRI is another forte of this study. Although our study is technically deemed as cross‐sectional, PI is a “fixed” variable, somewhat like one's genetic constitution that can allow prospective prediction as related to the phenotypes. However, our cohort represented Southern Chinese and additional studies are needed to assess the generalizability of our findings in other ethnic groups. Nonetheless, due to the homogeneity of our cohort, this further decreased any inherent confounds. Nonetheless, additional, prospective, and multi‐ethnic studies are further needed to validate our findings.

### 4.3 Clinical impact

Our findings further broaden the understanding of spine degeneration and, subsequently, pain. Currently, imaging techniques with even lower ionizing radiation dose exposure (eg, EOS) in comparison to conventional plain radiographs or no such exposure, such as ultrasound and smartphones, can assess one's spinopelvic alignment.^52^ Such information can obtain a more personalized profile of an individual, knowing who is at risk for spinal phenotype development. Such knowledge will allow early‐initiated and effective preventative measures and management options. This can facilitate more refined patient selection, for example in the context of biological regenerative therapies for the disc and/or endplate, as well as the development of novel, bespoke targeted therapeutics. In addition, the opportunity to identify unique variants/sub‐types of spinal phenotypes based on etiology and alignment patterns may shed light to facilitate more effective preventative and management protocols.

## 5 CONCLUSIONS

Though there are several previous studies who have seen the association of various spinal phenotypes on MRI and radiographic parameters of spinopelvic alignment^53^, ^54^ but our study is the “first” to identify that a low PI may “predict” the development of clinically‐relevant spinal phenotypes of lumbar disc displacement, MCs and UDS, irrespective of subject demographics and disc degeneration severity. A PI critical value of 42° or lower was found to have a fourfold increase in the development of these phenotypes. The association of PI with UDS is novel, stressing that altered biomechanics may induce disc calcification that can affect disc kinematics and the endplate. Such calcification can create stress concentrations leading to the initiation of annular fissures and cracks in the endplate, increasing the risk of MCs. Our study notes evidence for the “Evolutionary Etiology” pathway related to anterior column spinal phenotypes that may lead to LBP/sciatica **(**Figure 4 **)**. Understanding spinopelvic parameters and to potentially obtain one's “evolutionary spinopelvic imprint” with novel technology can lead to more personalized approaches to spine care and improved patient outcomes.

## CONFLICT OF INTEREST

The authors have no conflict of interest.

## AUTHOR CONTRIBUTIONS

U.Z. and D.S. conceived the study. C.B., J.C., D.S., and U.Z. collected data. U.Z. and D.S. performed the statistical analyses. U.Z. wrote the initial draft of the manuscript. D.S. and R.J.C. provided key edits to the manuscript. All the authors provided edits and revision of the manuscript. All authors interpreted the findings. D.S. obtained funding, supervised the study, and provided administrative support. All of the authors have read and approved the final submitted manuscript.
